# Table Olive Wastewater: Problem, Treatments and Future Strategy. A Review

**DOI:** 10.3389/fmicb.2018.01641

**Published:** 2018-07-23

**Authors:** Bárbara Rincón-Llorente, David De la Lama-Calvente, María J. Fernández-Rodríguez, Rafael Borja-Padilla

**Affiliations:** Department of Food Biotechnology, Instituto de la Grasa, Agencia Estatal Consejo Superior de Investigaciones Científicas (CSIC), Sevilla, Spain

**Keywords:** table olive wastewaters, advanced oxidation processes, biological treatments, bioremediation technologies, added value compounds

## Abstract

The table olive industry produces a high quantity of wastewater annually. These wastewaters are very problematic because of their characteristics of high organic matter, high phenolic content, high salinity and conductivity. The quantities in which they are produced are also a serious problem. The worldwide production of table olives reached 2,550,000 tons in the last five campaigns, with the European Union contributing to 32% of total production. The problem of these wastewaters is focused on the Mediterranean area where the highest quantity of table olives is produced and to a lesser extent on the United States and South America. Countries like Spain produce around 540,000 tons of these wastewaters. At present, there is no standard treatment for these wastewaters with acceptable results and which is applied in the industry. Currently, the most common treatment is the storage of these wastewaters in large evaporation ponds where, during the dry season, the wastewater disappears due to evaporation. This is not a solution as the evaporation ponds depend completely on the climatology and have a high number of associated problems, such as bad odors, insect proliferation and the contamination of underground aquifers. Different studies have been carried out on table olive wastewater treatment, but the reality is that at the industrial level, none has been successfully applied. New and promising treatments are needed. The current review analyzes the situation of table olive wastewater treatment and the promising technologies for the future.

## Introduction

The recent worldwide production of table olives was around 2.5–2.6 million tons (average data corresponding to harvest seasons 2011/2012 to 2016/2017, last season’s data are provisional) with a prediction for the 2017/2018 season of around 2.8 million tons. The table olive industry is an economic activity which is widely extended throughout the Mediterranean countries. The countries belonging to the European Union produce 886,500 tons (season 2015/2016) and other countries like Egypt, Turkey, Algeria, Syria, and Morocco have a total production of 1,223,500 tons (season 2015/2016), making them the main producers of table olives. High productions are also achieved by the United States (70,500 tons in season 2015/2016) and some South American countries like Argentina, Mexico and Peru with a production of 151,500 tons (season 2015/2016) (International Olive Council [IOC], 2017).

**FIGURE 1:**
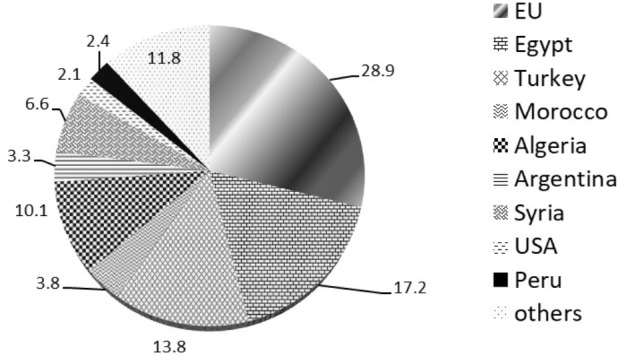
Global prediction for table olive production (in percentage of the overall predicted production of 2.8 million tons for season 2017/2018) by countries. Source: International Olive Council [IOC], 2017.

**FIGURE 2:**
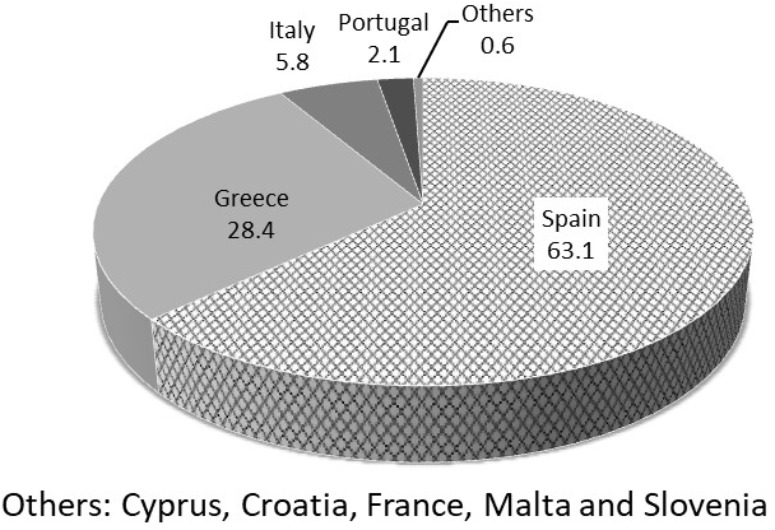
Prediction for table olive production in the EU (in percentage of the overall predicted production of 2.8 million tons for season 2017/2018) by countries. Source: International Olive Council [IOC], 2017.

**Figure 1** shows the global forecast for table olive production (in percentage of the overall predicted production of 2.8 million tons for season 2017/2018) by producing countries (International Olive Council [IOC], 2017). **Figure 2** shows the forecast for table olive production in Europe (in percentage of the overall predicted production of 2.8 million tons for season 2017/2018) by countries (International Olive Council [IOC], 2017).

The elaboration process of table olives results in the generation of a high volume of wastewaters coming from the various steps of industrial elaboration. The organic charge, chemical composition and the characteristics of the different streams produced during table olive processing vary depending on the preparation type. During the elaboration process of table olives different chemicals are used, e.g., NaOH, NaCl, lactic acid, etc., and high amounts of clean water are used for the de-bittering step, the different rinses, the brining and the packing step. The chemical characteristics and the volume of these wastewaters make them a huge environmental problem.

Olives which use lye for their preparation require multiple washes and can use up to five times the amount of potable water compared with natural methods. Furthermore, table olive processing using lye treatments has higher requirements of energy and labor costs (Kailis and Harris, 2007), but table olives elaborated in this way have shorter processing times than those elaborated by natural methods, and are in high demand around the world.

Generally, the effluents produced in each step of the elaboration process are mixed in one stream that is stored in evaporation ponds. This treatment technique is even more difficult than treating each effluent or wastewater separately. In addition, the storage in evaporation ponds of these wastewaters depends completely on climate and has a high number of associated problems like bad odors, insect proliferation and contamination of underground aquifers. In some countries these facilities are not allowed (Martin, 1992), but in several European countries, including Spain, they are still operational. Such practices are changing with the time in an attempt to care for the environment. In this sense, some of the solutions used in the process are now reused, such as the use or re-use of only one lye solution for de-bittering different batches of olives. Traditionally, the de-bittering step was carried out using fresh lye every time, but it has been proven that it is viable to use exhausted lye from other de-bittering steps, achieving lower environmental pollution and less water consumption (Garrido Fernández et al., 1997; Segovia-Bravo et al., 2008). With the same target in mind, the possibility of replacing the wash of the olives after the de-bittering step by re-using waters or the use of some organic and inorganic acids to neutralize the NaOH has also been studied (Garrido Fernández et al., 1997; Sánchez-Gómez et al., 2006).

Besides the fact that a huge volume of wastewater is produced, two of the main wastewaters, de-bittering and rising waters, are produced seasonally between September and November, due to the seasonal olive recollection (Ferrer-Polonio et al., 2017a). In addition, table olive processing is concentrated in narrow geographic areas where wastewater production is very high. This fact makes the situation even worse because of the huge volume of wastewaters generated in a short frame of time and place. Fermentation wastewaters from table olive processing, unlike the de-bittering and rising wastewaters, are generated during the year in the packaging plants. This fact and the very different characteristics of them, i.e., high pH and strong alkalinities in lyes and subsequent washing wastewater with acidic pH, oils in suspension, polyphenols and high salinity in brine wastewater, make it necessary to segregate these effluents from the general drainage systems in order to treat them separately (Romero-Barranco et al., 2001). A sustainable solution would be to refrain from mixing them. These practices would avoid the outlay of many liters of clean water.

The use of low concentration lyes or the re-use of fermentation brine are other practices studied (Garrido Fernández et al., 1997). Romero-Barranco et al. (2001) studied the possibility of introducing salt-free or reduced salt processes and segregation.

The direct re-use of fermentation wastewater or spent brines, coming from green or naturally black table olive processing has been studied. However, “the presence of metabolites interferes with the subsequent fermentation process” (Romero-Barranco et al., 2001). The presence of combined acidity, polyphenols, etc. does not provide the quality for safe storage and some organoleptic attributes of the olives can be damaged (Brenes et al., 1989; Romero-Barranco et al., 2001). The re-use of fermentation wastewater or partially regenerated brines has also been studied by Romero-Barranco et al. (2001).

Other measures studied aimed to better the sustainability of table olive processing by using acidified water instead of brines in the case of Californian-style black olives (olives darkened by oxidation) (De Castro et al., 2007), thereby reducing the NaCl concentration in the wastewater. The acidification of the media favors lactic acid bacteria and makes the media incompatible with enterobacteriaceae growth, with yeasts being the prominent microorganisms in these solutions (De Castro et al., 2007; Rejano et al., 2010).

The legislation in different countries concerning environmental issues is becoming more and more strict in order to control pollution. In addition to the separation of wastewaters and the improvement of operational procedures in the industry, wastewater treatment is also necessary. To increase the sustainability and reduce the environmental impact of the traditional table olive elaboration process different treatments for the wastewaters from table olive processing (TOPW) have also been studied and applied. Among the TOPW treatments studied there are several studies that use advanced oxidation processes (AOPs) such as: ozonation (Benítez et al., 2003), Fenton’s reaction (Kotsou et al., 2004), electrochemical treatments (Deligiorgis et al., 2008), TiO_2_ photocatalysis (Chatzisymeon et al., 2008), electro-coagulation (García-García et al., 2011) and wet air oxidation (Katsoni et al., 2008). Biological treatments have also been explored and include anaerobic digestion (Borja et al., 1993; Beltrán et al., 2008), aerobic digestion processes (Brenes et al., 2000; Benítez et al., 2002b) and combinations of the two (Aggelis et al., 2001; Ferrer-Polonio et al., 2015).

Several works employed bioremediation technologies using microalgae to remove pollution (Serrano et al., 2017) and others which use fungi obtained promising results for chemical oxygen demand (COD) removal from wastewater (Lasaridi et al., 2010) and for de-colorization (Ayed et al., 2016). Other approaches have been to use certain wastewaters for irrigation (Murillo et al., 2000) or for the extraction and recovery of added-value products (Brenes et al., 2004; Kiai et al., 2014).

This review analyzes the current situation of the treatment for wastewater from table olive processing and gives an overview of the different strategies and treatments studied along with promising technologies for the future.

## Table Olive Elaboration Process

The table olive elaboration process starts after picking the olives from the olive tree (*Olea europaea L.*) when they have a good size and color, e.g., from green to yellow. After picking, leaf removal and classification, the table olive elaboration process follows three main steps:

- *De-bittering or lye step*: in this step olive bitterness is removed by immersing the olives in a NaOH aqueous solution with concentrations between 1 and 2% w/v during 8–12–15 h (Parino et al., 2007; Cappelletti et al., 2011). The concentration of the NaOH used depends of the olive variety, the degree of ripeness of the drupe and the temperature and characteristics of the water to be used. More concentrated solutions can soften the flesh of the drupe, while more dilute solutions adversely affect the subsequent fermentation (Cappelletti et al., 2011). In this first step oleuropein is hydrolyzed to elenolic acid glucoside and hydroxytyrosol (Marsilio and Lanza, 1998; Marsilio et al., 2001).
- *Rinsing*: after the de-bittering step, the olives are washed, one or more times until all the alkali is removed (Fendri et al., 2013). This step uses large quantities of fresh water to separate the sodium hydroxide from the flesh of the olives and can vary in duration. The most commonly used method for washing is to rinse for 18–25 h with an initial short rinse of 1–2 h and two more rinses of 8–12 h each. “In this case the olives retain enough fermentable substances to ensure proper lactic fermentation” (Cappelletti et al., 2011). There are other options for longer or shorter duration rinses depending on the purpose of the olives to be washed.
- *Fermentation in brine*: after the rinsing step, the olives are submerged in a 9–10% w/v concentration NaCl. Fermentation preserves the olives and improves their organoleptic properties (Marsilio and Lanza, 1998). The addition of used brines or “mother brines,” ensures the onset of a safe lactic fermentation (Cappelletti et al., 2011).

Finally, after the olives are washed with new water and after selection, avoiding damaged ones, they are packaged in 3–5% brine and pasteurized at 90°C during 1 h, following traditional elaboration processes.

**FIGURE 3:**
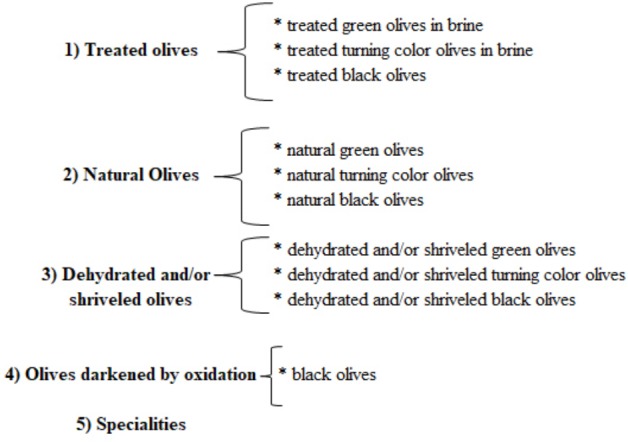
Table olives sorted regarding standard trade preparations (International Olive Oil Council [IOOC], 2004).

**FIGURE 4:**
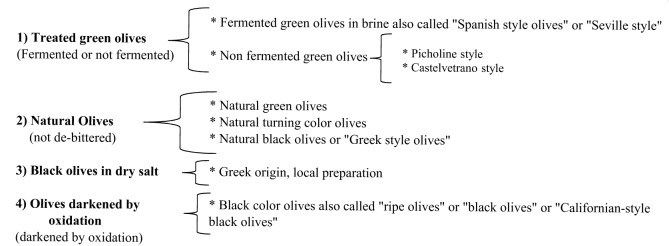
Table olives sorted regarding the economic importance from global standpoint (adapted from Rejano et al., 2010).

There are many elaboration processes for table olives depending of the kind of olives and their specific necessities according to their natural composition, degree of ripeness, country of origin, local or regional customs, etc. Regarding standard trade preparations table olives can be sorted as shown in **Figure 3**. The most important processing methods regarding economic importance from a global standpoint are shown in **Figure 4** and detailed below

### Treated Green Olives

“Treated olives are green olives, turning color olives or black olives that have undergone alkaline treatment, then placed in a brine where they undergo complete or partial fermentation, and are preserved or not by the addition of acidifying agents” (Sánchez-Gómez et al., 2006).

Green olives are harvested when they reach an average size, prior to color variation and are usually picked manually. The main treatments used for green olive preparation are: fermentation or “Spanish-style olives” and non-fermentation or “Picholine and Castelvetrano styles” (Sánchez-Gómez et al., 2006; Rejano et al., 2010).

#### Fermented Treated Green Olives or “Spanish-Style Olives”

After harvesting, transport and grading, green olives are kept in a lye solution (2.0–3.5% w/v, NaOH in water). Treatments usually take place in 10-ton capacity containers “until the lye has penetrated 2/3 of the way through the flesh” (Rejano et al., 2010). When the lye has reached this depth it is replaced by water to eliminate all the alkali in different wash steps and “to drag over soluble sugars needed for fermentation” (Rejano et al., 2010). Finally, the olives are preserved in brine (9–10% NaCl initially) which propitiates the culture media for fermentation. Brine concentration typically drops to 5% owing to the interchange between the water and the olives.

Initially, only Enterobacteriaceae, Gram-negative bacteria and Enterococci grow and they gradually become undetectable when the pH decreases “as a consequence of their own metabolism” (De Castro et al., 2002). Acid generation by these microorganisms favors the growth of lactobacilli, which is mainly responsible for fermentation (De Castro et al., 2002).

#### Non-fermented Treated Green Olives or “Picholine Style” and “Castelvetrano Style”

“Picholine style” for table olive preparation is used in the south of France, Morocco and Algeria. Picholine table olives undergo an initial de-bittering step with lye (2.0–2.5% NaOH w/v) for 8–12 h. After this time they are washed and placed in brine (5–6%) for 2 or 3 days, and then changed to a more concentrated brine (7%), using citric acid to keep the pH at around 4.5. The olives are ready for use after 8–10 days (Rejano et al., 2010).

“Castelvetrano style” for table olive preparation is used in the Castelvetrano region, Italy, with the olive variety “Nocellara del Belice.” After harvesting, the olives are placed in a NaOH solution (1.8–2.5% w/v) with 5–8 kg of salt/140 kg of fruits 1 h after the de-bittering step begins. The olives are kept in this solution for 10–15 days. After this period, a light washing takes place before consumption (Salvo et al., 1995; Rejano et al., 2010).

### Natural Olives

Natural olives are green olives, turning color olives or black olives that are placed directly in brine where they undergo complete or partial fermentation, preserved or not by the addition of acidifying agents (Sánchez-Gómez et al., 2006).

Natural olives are not de-bittered, they are placed directly in brine (9% NaCl) when they arrive to the table olive factory and they undergo a fermentation process. At the beginning of fermentation, the tanks are hermetic to avoid contact with air and to maintain anaerobic conditions. This kind of fermentation “takes a long time because the diffusion of soluble compounds through the epidermis in fruits not treated with alkali is slow” (Rejano et al., 2010). Natural olives are prepared from dark olives, but they are also prepared from green. Yeasts are the main microorganisms in this kind of brine although there are diverse microorganisms. Although the olives are not treated with lye, the brine reduces their bitterness and the olives are not packaged until the bitterness is weak enough. The color of the olives is corrected after fermentation by aeration or treatment with ferrous gluconate or lactate. “Natural black olives” are also known as “Greek style olives” (Sánchez-Gómez et al., 2006).

### Black Olives in Dry Salt

This preparation uses overripe olives and has Greek origin. For this preparation, the olives are placed in baskets and covered with layers of salt (15% of the weight of the olives), and usually used for local consumption (Rejano et al., 2010).

### Olives Darkened by Oxidation

Olives darkened by oxidation are green olives or turning color olives preserved in brine, fermented or not, darkened by oxidation in an alkaline medium and preserved in hermetically sealed containers subjected to heat sterilization; “they shall be a uniform black color” (International Olive Oil Council [IOOC], 2004). These are also known as “ripe olives” or “black olives” (Sánchez-Gómez et al., 2006) or “Californian-style black olives.”

Californian-style black-ripe olives are olives treated and oxidized during the elaboration process to produce ranges of color from dark brown to black. These olives are picked when they are partially or completely ripe and then they are stored in brine (8–10%) during 30 days before treatment with NaOH (Sánchez-Gómez et al., 2006).

The de-bittering step is usually carried out with a NaOH (1–2% w/v) solution, applying at least three de-bittering treatments in a row, during 2 to 6 h. After each pretreatment there is a rinse step where air is bubbled into the water, producing an enzymatic reaction which causes the surface of the olives to darken (Brenes et al., 2004; Sánchez-Gómez et al., 2006). The lye treatments are between three and five. The main objective is to achieve a gradual penetration of the lye into the flesh so that it reaches the pit (Rejano et al., 2010).

Finally, the olives are washed to remove the sodium hidroxyde until a pH in the flesh of 8.0. Olives darkened by oxidation have to be sterilized to avoid pathogen growth.

## Table Olive Processing Wastewaters (TOPW): Chemical Composition and Volumes Produced

Although there are three main streams that are produced after table olive processing, such as wastewaters coming from lye, wastewaters coming from the washes and wastewaters from fermentation brines, there are additional wastewaters from a table olive processing plant which are produced from the washing of the plant, cleaning of the vessels or containers, etc. There also are many different kinds of wastewaters produced, depending on the elaboration process, e.g., Spanish-style green olives, Californian-style, etc., the degree of maturation of the olives, the kind of water employed, and the additives used, among other factors. All of the wastewaters from table olive processing cause a serious environmental problem because of their chemical characteristics and the huge volumes produced. Lye wastewaters and the subsequent washing wastewaters are so problematic because of their high pH and strong alkalinity, and brine wastewaters because of their acidic characteristics and salinity, with high ClNa concentrations, as well as the organic charge due to the interchange of compounds with the olives during table olive processing, as in treated and fermented green olives where the content in polyphenols in the wastewaters coming from fermentation brines is rich but null in reducing sugars which are consumed in the fermentation step.

The volumes of wastewaters produced during the table olive processing methods are shown in **Table 1**. The most polluting effluents produced are those that include a lye treatment followed by exhaustive washings for the elimination of the alkali. Among them the production of Californian-style black-ripe olives has the highest pollutant potential with around 2–6 L/kg olives produced (Garrido Fernández et al., 1997; Papadaki and Mantzouridou, 2016), followed by the Californian green ripe olives and Spanish table olives with an average of 1.5–3.5 L/kg olives produced and finally, the Naturally black olives and the untreated green and turning color olives with 1 L/kg olives produced. Wastewaters from fermentation in brine represent 20% of the total volume within the global industry; it is 85% of the global wastewater pollution (Garrido Fernández et al., 1997; Moussavi et al., 2010; Ferrer-Polonio et al., 2016a).

**Table 1:** Volume of the different effluents produced during table olive processing (lye, fermentation brine, washing, and preservation brine) by the different methods in Liters/kg of table olives.

	Spanish style	Untreated green and turning color olives	California green ripe olives	California black ripe olives	Naturally black olives
(1) Lye	0.5		0.5	0.5–0.25	
(2) Fermentation brine	0.5	0.5	0.5	0.5	0.5
(3) Washing	0.5–2.0		0.5–2.0	0.5–3.0	
(4) Preservation brine	0.5	0.5	0.0–0.5	0.5	0.5


*Source: Garrido Fernández et al., 1997.*

**FIGURE 5:**
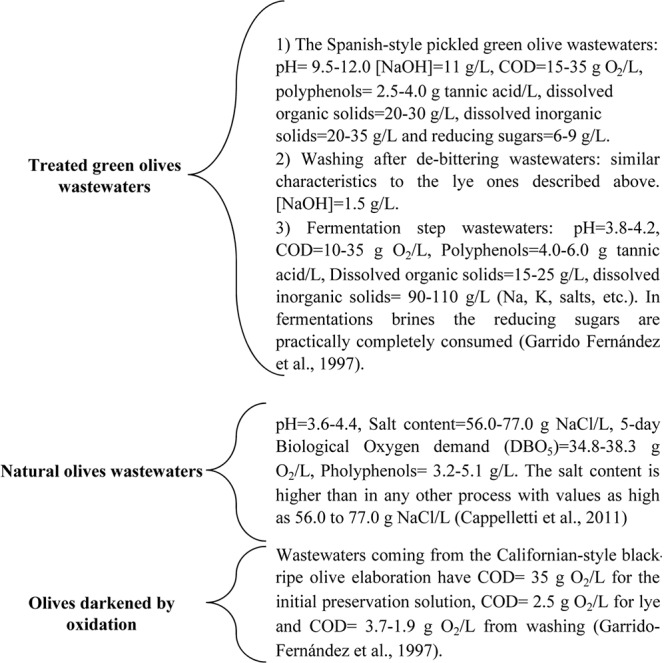
Main characteristics of the generated from the most economically important elaboration systems.

**Figure 5** shows the main characteristics of the generated wastewaters from the most economically important elaboration systems.

## Table Olive Processing Wastewater Treatments

### Advanced Oxidation Processes (AOPs)

Advanced oxidation processes have been presented as adequate methods for treating TOPW due to their ability to reduce the organic matter content.

The main systems used as AOPs are ozonation, UV irradiation, photocatalysis, hydrogen peroxide oxidation, Fenton’s reaction, electrochemical oxidation and wet air oxidation.

These processes are characterized by the generation of highly reactive free radicals which are capable of oxidizing several organic substances, such as phenols. These compounds are able to react with carbon–carbon double bonds and thus attack the aromatic nucleus, which are typical characteristics of refractory organic compounds (Zaviska et al., 2009).

One of the main issues with these processes is their high operational costs (Comninellis et al., 2008), thus the application of AOPs for treating TOPW is only recommended when a biological process is not possible or is insufficient. **Table 2** summarizes the operating conditions, process efficiencies and benefits derived from the use of AOPs for the treatment of these wastewaters.

**Table 2:** Summary of the operating conditions, process efficiencies and benefits derived from the use of advanced oxidation processes (AOPs) for TOPW treatment.

Wastewater type	Treatment	Operating conditions	Process efficiency	Benefits	Reference
Green olive de-bittering wastewaters	Ozonation	O_3_: 10g. O_3_ rate: 7⋅10^-4^ mol min^-1^. Time: 5 h. COD_0_: 20 g O_2_/L.	COD removal: 50%.	The pH was stabilized from 13 to 9.6.	Beltrán et al., 1999
Black olive washing wastewaters	Ozonation	Time: 3 h. Temperature: 10–30°C.	COD removal: 24–33%.	An increase in temperature enhanced the COD and aromaticity removal.	Beltrán-Heredia et al., 2000b
Table olive processing wastewaters	Ozonation	[O_3_]: 45 mg O_3_/L. O_3_ rate: 20 L/h. Time: 35 min.	COD removal: 80% (after aerobic digestion).	This treatment enhanced the post aerobic digestion, by reducing the pH, the phenols content and the ammonia.	Rivas J. et al., 2000
Green olive de-bittering and washing wastewaters diluted with urban wastewaters	Ozonation	O_3_-O_2_ rate: 0–20 L/min. Acidic and basic cycles (pH 4–10). Time: 3 h.	COD removal: 80%.		Rivas F.J. et al., 2000
Black olive washing wastewaters	Ozonation	P(O_3_): 4.25 kPa. O_3_-O_2_ rate: 0–40 L/min. Temperature: 20°C. Time: 6 h.	COD removal: 80%.	The pH was stabilized	Benítez et al., 2001
Black olive lye-wastewaters	Ozonation	P(O_3_): 2.76–4.36 kPa. Temperature: 20°C. Time: 8 h.	COD removal: 70%.	An increase in initial ozone pressure increased phenol removal	Benítez et al., 2002a
Black olive processing wastewaters	Ozonation	P(O_3_): 1.04 – 4.5 kPa.	COD removal: 14–23%.	COD removal depends on the initial ozone pressure.	Benítez et al., 2003
Green olive de-bittering wastewaters	Ozonation + H_2_O_2_	O_3_: 3–4 g_._ O_3_ rate: 5⋅10^-4^ mol min^-1^. [H_2_O_2_]: 10^-3^ M_._ Time: 2 h.	COD removal: 80–90%.	Addition of H_2_O_2_ enhanced the COD removal from 50% to 90%.	Beltrán et al., 1999
Black olive washing wastewaters	Ozonation + H_2_O_2_	P(O_3_): 4.25 kPa. O_3_-O_2_ rate: 0–40 L/min. [H_2_O_2_]: 0.0525–0.129 M. Temperature: 20°C. Time: 6 h.	COD removal: 92%.	Addition of H_2_O_2_ enhanced the COD removal from 80 to 92%.	Benítez et al., 2001
Black olive processing wastewaters	Ozonation + H_2_O_2_	P(O_3_): 4.5 kPa [H_2_O_2_]: 0.2–0.5 M	COD removal: 24–29%.	Addition of H_2_O_2_ enhanced the COD removal from 23 to 29%.	Benítez et al., 2003
Green olive de-bittering wastewaters	Ozonation + UV	O_3_: 3–4 g. O_3_ rate: 6.91⋅10^-4^ mol min^-1^. UV: 254 nm. Time: 2 h.	COD removal: 80–90%.	UV addition enhanced the COD removal, although, using H_2_O_2_ the TC depletion is higher (55%).	Beltrán et al., 1999
Black olive washing wastewaters	Ozonation + UV	P(O_3_): 4.25 kPa. O_3_–O_2_ rate: 0–40 L/min. UV: (Hanau TQ150 high-pressure mercury vapor lamp) 1.76 ⋅ 10^-5^ einstein/s. Temperature: 20°C. Time: 6 h.	COD removal: 92%.	Using UV enhanced the aromatic compound removal when comparing with single ozonation or H_2_O_2_ addition.	Benítez et al., 2001
Black olive lye-wastewaters	Ozonation + UV	P(O_3_): 4.41 kPa. Temperature: 20°C. Time: 8h.	COD removal: 85%.	The use of UV enhanced polyphenol removal (100%).	Benítez et al., 2002a
Black olive processing wastewaters	Ozonation + UV	P(O_3_): 1.04 – 4.5 kPa.	COD removal: 16–39%.	Using UV enhanced COD removal when comparing with single ozonation or H_2_O_2_ addition.	Benítez et al., 2003
Black olive processing wastewaters	Ozonation + H_2_O_2_ + UV	P(O_3_): 4.5 kPa. [H_2_O_2_]: 0.2–0.5 M.	COD removal: 39%.		Benítez et al., 2003
Black olive processing wastewaters	Fenton’s Reagent + UV	Temperature: 20°C. Time: 8 h. [Fe^2+^]: 0.025M. [H_2_O_2_]: 0.5M.	COD removal: 24%.		Benítez et al., 2003
Table olive washing and de-bittering wastewaters	Fenton’s reagent	[H_2_O_2_]: 2, 4, 6, 8 g/L.	COD removal: 34%.	The pH was reduced to 2.2	Kotsou et al., 2004
Black olive washing wastewaters	Electrochemical Treatment (BDD)	COD_0_: 10 g O_2_/L. Current intensity: 30 A. Time: 14 h.	COD removal: 73%.	Initial pH and H_2_O_2_ did not show any enhancement in COD removal.	Deligiorgis et al., 2008
Meski olive washing and de-bittering wastewaters	Electrochemical Treatment (BDD)	Time: 2 h. Current density: 110 mA/m^2^.	COD removal: 97%.		Gargouri et al., 2017
Black olive processing wastewaters	Electrochemical Treatment (BDD)	SBR mode: 0.5 L/min. COD_0_: 7500 mg O_2_/L. Time: 30–240 min. Current density: 187.5 mA/cm^2^.	COD removal: 96.5%.	The pH was stabilized	Tatoulis et al., 2016
Meski olive washing and de-bittering wastewaters	Electrochemical Treatment (Led dioxide electrode)	Time: 2 h. Current density: 110 mA/m^2^.	COD removal: 71%.		Gargouri et al., 2017
Green olive washing and de-bittering wastewaters	Electrochemical Treatment (Fe electrode)	[H_2_O_2_]: 17 g/L.	COD removal: 75%.	The use of Ca(OH)_2_ enhanced the COD removal up to 98%.	Kyriacou et al., 2005
Table olive processing wastewaters	Electrochemical Treatment (Planar graphite electrode) + Anaerobic Digestion	Temperature: 35°C. Current density: 7.1 A/m^2^.	COD removal: 32%. Maximum methane yield: 109 NmL CH_4_/g COD.	This wastewater did not produce methane during the anaerobic digestion when the potential was not applied.	Marone et al., 2016
Black olive de-bittering wastewaters	Photocatalysis (TiO_2_)	COD_0_: 1–8 g O_2_/L. UV-A: 300–366 nm.	COD removal: 13–38%.	An addition of H_2_O_2_ enhanced the COD removal by 20%.	Chatzisymeon et al., 2008
Green olive processing wastewaters	Electro-coagulation (Al/Fe)	Temperature: 20-25°C. Time: 50 min. Current density: 25 mA/cm^2^.	COD removal: 40%.	The pH was neutralized.	García-García et al., 2011
Green olive washing and de-bittering wastewaters	Wet air oxidation	P(O_2_): 5 MPa. Time: 6–8 h. [Cu^2+^]: 419.4 mg/L. Temperature: 180°C.	COD removal: 59.8%.	Reducing the amount of Cu^2+^ enhanced the phenol depletion up to 95%, although, COD removal was lower (28.5%).	Rivas et al., 2001
Black olive fermentation wastewaters	Wet air oxidation	P(O_2_): 2.5 MPa. COD_0_: 1240 mg O_2_/L. Temperature: 180°C. pH: 7. Time: 2 h.	COD removal: 70%.	Reaction time, temperature and initial pH affected significantly the COD reduction.	Katsoni et al., 2008

*COD_0_: initial COD.*

#### Ozonation

Ozonation consists of the use of ozone, a powerful oxidant which can either decompose in water, forming hydroxyl radicals that act as a stronger oxidant, or attack functional groups of organic compounds through an electrophilic mechanism (Ayed et al., 2017). The main mechanism of these advanced oxidation processes consists of the production of highly free radicals which can react with phenols through aromatic substitution and/or dipolar cyclo addition reactions (Langlais et al., 1991). Commonly, this treatment is enhanced using hydrogen peroxide as a further oxidant and UV radiation as a photocatalytic agent. Generally, these three oxidant agents can directly oxidize organic matter, though several studies show that some positive synergetic effects take place between them. Ozone in the presence of H_2_O_2_ generates hydroxyl radicals and in the presence of UV radiation ozone can be converted into more hydrogen peroxide which produces hydroxyl radicals and increases its oxidant activity. Thus, when these agents are used together the main oxidative agents are the hydroxyl radicals (Benítez et al., 2003).

Single ozonation was applied to wastewaters derived from the de-bittering step of green table olive processing for 5 h (with a COD of 21 g O_2_/L) with a COD removal of nearly 50% (Beltrán et al., 1999). After ozonation the pH was reduced from 13 to 9.6; aromatic compounds were reduced by 23% and the color was completely eliminated (Beltrán et al., 1999).

A further study by Beltrán-Heredia et al. (2000a) evaluated the kinetics of the aerobic biological processing of black table olive wastewaters using the Contois model. Results showed that a single aerobic treatment led to specific kinetic parameters for the substrate removal rate (COD and total phenols) of 4.81⋅10^-2^ h^-1^; a cellular yield coefficient of 0.279 g VSS/g COD (VSS: volatile suspended solids), and finally, the kinetic constant for endogenous metabolism was 1.92⋅10^-2^ h^-1^. When ozonation was applied prior to the aerobic treatment these parameters were: 5.42⋅10^-2^ h^-1^, 0.280 g VSS/g COD and 9.1⋅10^-3^ h^-1^, respectively. So the use of ozone as a previous step before aerobic degradation improved the kinetics of the process as well as the pollutant reduction, as reported in this previous work.

Ozonation processes for wastewater resulting from the de-bittering stage of green table olive preparation have been improved by the combination with hydrogen peroxide and/or UV radiation. For instance, an 80% or 90% COD removal was achieved with ozone doses of 3 and 4 g with the addition of hydrogen peroxide (10–3M) or UV radiation (254 nm) (Beltrán et al., 1999).

An ozone dosage of 45 mg/L (flow rate 20 L/h; 35 min) decreased the pH (from 11.5 to 7.5–8), phenol content (35%) and nitrogen as ammonia (70%) in table olive wastewater with an increase in biodegradability which allowed for an 80% COD removal after aerobic digestion (Rivas J. et al., 2000).

Rivas F.J. et al. (2000) showed that using ozonation as a first step followed by the aerobic treatment of green table olive wastewaters generated in the de-bittering and washing steps of this process and diluted with urban wastewaters (final COD: 1450 mg O_2_/L) increased biodegradability by 100% (as BOD_5_/COD ratio) when acidic and basic cycles (pH 4 and 10) were applied in the ozonation step. This combined treatment led to a 90% phenolic compound removal and 80% COD removal during the ozonation step, and a further reduction in COD by 38% after the aerobic process.

When the ozonation of black table olive wastewaters from the washing step was applied after aerobic digestion an 87% COD removal was obtained, which implies that aerobic pre-treatment improved subsequent ozone action due to the elimination of most of the organic matter. Thus, ozone applied after aerobic degradation acted mostly on non-biodegradable compounds while the phenolic compounds were basically removed in the previous aerobic stage (Beltrán-Heredia et al., 2000b).

When ozonation was applied to black table olive wastewater from the washing step an 80% substrate removal was reached. Moreover, a 78% aromatic compound removal and a reduction in pH from 12.62 to 8.26 were accomplished when the inlet ozone pressure was 4.25 kPa and applied for 6 h (Benítez et al., 2001). A combined ozonation with hydrogen peroxide or UV-radiation has been also studied and the results showed an enhancement in substrate and aromatic compound removals. When combined with hydrogen peroxide a maximum substrate removal was reached (92%), although when combined with UV radiation a better aromatic compound removal (100%) and lower pH (8.54) were observed (Benítez et al., 2001). In this same study, ozonation (4.20 kPa; 20°C; 2 h) after aerobic biodegradation (3.75 days) was evaluated, and this combined treatment reduced substrate concentration by 90%, which could be enhanced when UV radiation was coupled with ozone, reaching a total substrate reduction of 93%.

Lye wastewaters produced during the de-bittering and darkening of black table olives have also been studied due to their alkali contents. Benítez et al. (2002a) showed that single ozonation of this lye wastewater (20°C, pH 13.6 and 8 h) reduced the COD by 70%. This study also showed that raising the initial pressure of the ozone from 2.76 to 4.36 kPa affected COD removal by only 2%, while polyphenol removal increased from 87 to 94%. pH was also affected during single ozonation and was reduced to 9.89 and 9.28, depending on the initial ozone pressure; while aromaticity removal was not affected by initial ozone pressure (80%). In this study some combinations of O_3_ with H_2_O_2_ or UV radiation were also evaluated. The results showed that the best combination resulted from O_3_ plus UV radiation with an initial ozone pressure of 4.41 kPa which led to an 85% COD removal, a 99% aromaticity removal, a 100% polyphenol removal and a decrease in pH to 9.32.

Benítez et al. (2003) evaluated the effect of ozonation combined with H_2_O_2_ and UV radiation on wastewaters from black table olive processing. This study concluded that single ozonation (1.04–4.48 kPa) reduced COD concentrations by 14–23%, depending on the initial ozone pressure and aromatic compounds by 73%, regardless of the initial O_3_ pressure. The combination of O_3_ (4.5 kPa) and H_2_O_2_ (0.2–0.5 M) resulted in a COD removal of 24–29% and an aromatic reduction of 74–75%. A combined ozone (1.04–4.50 kPa) and UV radiation treatment reached a 16–39% COD removal and aromatic reduction of 83–86%. When H_2_O_2_ and UV radiation were used in combination with O_3_ the COD removal increased by up to 39% and the aromatic reduction to 86%. Single UV radiation reached COD and aromatic compound removals of 9 and 27%, respectively. These removals increased when 0.5 M of H_2_O_2_ was used (COD removal increased to 13% and the aromatic removal to 38%).

Moreover, Benítez et al. (2003) studied the effect of ozonation and ozonation/UV radiation as a pre-treatment for aerobic degradation. A single aerobic treatment (initial concentration from 0.54 to 3.55 gVSS/L; initial COD = 34.2 g/L) led to a COD removal of 66–67%, which can be enhanced when ozone (3.04 kPa; 2 h) and ozone/UV were used previously, which led to a COD removal of 71% in both cases.

In most cases, the ozonation of table olive wastewater has proven to be an excellent pre-treatment for further biological processing such as aerobic digestion due to its capacity for phenolic compound removal, alkalinity depletion and pH reduction. Moreover, COD removals of 80–90% were achieved.

However, the major limitation of this process was the high cost of ozone generation coupled with its short half-life period (Ayed et al., 2017). Another drawback was the low solubility of O_3_ in these solutions which reduced its efficiency (Gogate and Pandit, 2004).

#### Fenton’s Reaction

Fenton’s reaction consists of the addition of H_2_O_2_ and Fe (II) salts directly into wastewaters. The oxidation mechanism consists of the catalytic decomposition of H_2_O_2_ into hydroxyl radicals which produce an oxidized iron (III) which can act as a coagulation and sedimentation agent for other compounds. Moreover, these new compounds can also oxidize more hydrogen peroxide as well as phenolic compounds (Rivas et al., 2003; Cañizares et al., 2007). Furthermore, iron forms can produce several organic and inorganic complexes which highly affect the reactivity of this metal over hydrogen peroxide. Thus, iron complexes from carboxylic acids have been proven to act as accelerating agents, while phosphates inhibit the oxidation process (Rivas et al., 2003).

Benítez et al. (2003) evaluated the effect of Fenton’s reagent combined with UV radiation by treating wastewaters from black table olive processing. The results showed that at a temperature of 20°C during 8 h the COD removal reached 24% under the best conditions (i.e., [Fe^+2^] = 0.025M; [H_2_O_2_] = 0.5M).

A combined aerobic and Fenton’s reagent treatment of table olive washing waters and de-bittering processes was studied by Kotsou et al. (2004). After the aerobic stage (2-days), using *Aspergillus niger*, COD reduction was 70% and total and simple phenol depletions were 41% and 85%, respectively. During the oxidation step using Fenton’s reagent the effect of H_2_O_2_ concentration was evaluated. It was concluded that different concentrations (2, 4, 6, 8 gH_2_O_2_/L) did not show any effect on phenol removal, which is the first organic compound to be oxidized (within the first 15 min). Only the lowest concentration did not reach the same phenol removal due to the complete elimination of hydrogen peroxide. Other organic compounds were reduced by the hydrogen peroxide left. After the Fenton’s reagent treatment, COD removal was 34–72% (depending on H_2_O_2_ initial concentration), total phenolic compound removal was 64–91%, pH was reduced to 2.2 and an increase in temperature was observed to up to 34°C (Kotsou et al., 2004).

In order to reduce the operational cost of this AOP the replacement of ferrous iron by the ferric form could be an alternative (Ayed et al., 2017). One major disadvantage of this AOP is the necessity of a further treatment which may reduce the iron present in the effluent. In addition, the presence of hydrogen peroxide at the end of the treatment process can limit the efficiency of a further biological process (Gogate and Pandit, 2004). In addition, lower COD removals (24–34%) were achieved compared to Ozonation processes.

#### Electrochemical Treatment

The high conductivity of boron-doped diamond (BDD) and other types of electrodes makes these materials a good choice for electrochemical treatments which consist of the oxidation of H_2_O at the anode to generate adsorbed hydroxyl radicals in the electrode surface. These hydroxyl radicals are capable oxidizing organic compounds near the electrode zone into CO_2_ and H_2_O (Alvarez-Pugliese et al., 2014).

Wastewaters from the washing process of black table olives were treated using BDD electrodes with the aim of evaluating the effect of initial COD (1340–5370 mg O_2_/L), reaction time (30–120 min), current intensity (5–14 A), initial pH (3–7) and the application of hydrogen peroxide (500 mg/L) as an additional oxidant (Deligiorgis et al., 2008). After the study, it was concluded that the initial pH and the use of hydrogen peroxide did not present any effect on COD and total phenol removal. By performing a further experiment under the best obtained conditions (initial COD: 10 g O_2_/L; 30 A; 14 h) a reduction of 73% COD was achieved. Certain issues arose: the current intensity was near to the maximum recommended by the fabricator (35 A) and the higher the initial COD concentration the higher the temperature, which could turn out to be impermissible for the reactor.

Gargouri et al. (2017) compared lead dioxide electrodes and BDD electrodes as the anodes when treating Meski olive wastewaters from washing and de-bittering processes. The results showed that the pollutant reduction in this wastewater when using BDD electrodes presented a higher oxidation rate than that obtained with the lead dioxide electrode. Under the best conditions (2 h, 110 mA/m^2^), the BDD electrode presented a COD removal of 97% while lead dioxide electrode showed a 71% COD removal. This difference was explained on the basis of the different natures of the physio-sorbed hydroxyl radicals generated on both electrodes.

Kyriacou et al. (2005) studied the combined treatment of the wastewaters from green olive table washing and de-bittering processes by aerobic degradation and further electrochemical treatment with hydrogen peroxide. Aerobic degradation was performed using an *Aspergillus niger* strain as inoculum and ran for 3 days. The electrochemical system evaluated three different electrode types (iron, stainless steel and Ti/Pd) and several reaction times (30 and 60 min). The results showed that the aerobic degradation reduced the COD by up to 66–86% and the phenol compounds by up to 65%; pH was also reduced from 5.0 to3.5. The electrochemical treatment at laboratory-scale reduced the COD and phenol content by 97% when 2.5% H_2_O_2_ and an iron electrode were used for 60 min; while at pilot scale COD removal reached 75% when 1.7% H_2_O_2_ was used.

A combined aerobic system and electrochemical oxidation using the BDD treatment of black table olive wastewater was evaluated by Tatoulis et al. (2016). In this research the effects of the initial COD concentration (5500 – 15000 mg O_2_/L) and the operation mode [batch and sequential batch reactors (SBR) with recirculation] of the aerobic process on final purification were assessed. The results showed that the best conditions were obtained with SBR mode (recirculation: 0.5 L/min) and an initial COD of 7500 mg O_2_/L which resulted in a COD reduction of 96.5%, a phenol reduction of 64.5%, and a pH neutralization from 4 to 6 when an inoculum made from the original indigenous microorganism from wastewater was added at the beginning of the aerobic process. However, color could not be removed completely. When the BDD electrode was used together with the aerobic process the COD, phenol and color removal was complete with a current density of 187.5 mA/cm^2^ within 30–240 min, depending on the initial COD.

A different electrochemical system was used by Marone et al. (2016), in which a planar graphite plate was selected as working electrode in a hermetic potentiostatically controlled half-cell system in combination with an anaerobic digestion system at 35°C. Using table olive brine processing wastewater without any potential applied, no methane production was recorded in its anaerobic digestion. However, when a potential was applied a maximum methane yield of 109 ± 21 NmL CH_4_/g COD removed was observed with a current density of 7.1 ± 0.4 A/m^2^ and a coulombic efficiency of 30%. In addition, 80% of the phenolic compounds were removed, though COD was only reduced by 32%. Furthermore, a microbial study of anodic biofilms was performed by the sequencing of bacterial 16s rDNA, and no archaea was found and within the bacterial community the *Proteobacteria* were predominant (>48%) over *Bacteroidetes* and *Firmicutes*. The most abundant anode-respiring bacteria (ARB) found in every assay performed was *Desulfuromonas desulfuromonadaceae* (23–55%). Another predominant ARB found in some assays was *Geoalkalibacter geobacteraceae* (20–40%).

The electrochemical treatments are generally high cost processes, i.e., BDD electrode electricity costs can reach 7–10/kg COD removed (Chatzisymeon et al., 2009). They do not allow the total color and phenolic compounds removals from these wastewaters, for which further biological processes are needed. By contrast, acceptable COD reductions (75–95%) can be achieved.

#### TiO_2_ Photocatalysis

Photocatalysis consists of the excitation by UV or Visible of a semiconductor. The semicor (TiO_2_) transforms photon energy into chemical energy by redox reactions which produce activated sites of TiO_2_ and the subsequent degradation of organic compounds due to chain reactions promoted by strong radical oxidants like the OH generated by water molecules (Linsebigler et al., 1995).

Wastewaters from the de-bittering step of black table olives were used for photocatalysis treatment after dilution with distilled water to reach an initial concentration of 1–8 gO_2_/L (Chatzisymeon et al., 2008). In this study several commercial TiO_2_ were evaluated due to their different particle size and specific area, although UV-A radiation between 300 and 366 nm was kept the same for each experiment. The highest COD reduction (38%) was obtained with the TiO_2_ with the highest specific area, though total phenols and color reduction were almost the lowest (11 and 12%, respectively). The highest phenol removal (58%) was obtained with a TiO_2_ with a medium value of specific area when COD reduction was very low (13%) but color reduction was slightly high (77%). Then, the highest color reduction (83%) was observed with the second highest specific area TiO_2_. In each case, the TiO_2_ form was anatase, which presented a higher photocatalysis activity than the rutile form.

In a further study, Chatzisymeon et al. (2008) compared the same anatase TiO_2_ when the initial COD was changed (from 2 to 8gO_2_/L). This study concluded that the lower the initial COD concentration, the higher the reduction in COD, total phenols and color. Moreover, an improvement of 20% in COD, aromatic compounds and color removal was achieved when hydrogen peroxide was added during the treatment, although total phenol reduction remained unchanged.

The main advantage of TiO_2_ is its non-toxicity, water insolubility, hydrophobicity, cheap availability and photo-corrosion stability nature. However, a great drawback is the necessity of UVA irradiation for a good photoactivation. Since solar irradiation into the earth’s surface only contains 3–5% of UVA, an enhanced method needs to be studied (Comninellis et al., 2008). Moreover, the effluents obtained using this method, even when the organic content has been reduced, present less biodegradable compounds than the untreated wastewater as can be seen when table olive wastewaters are treated only aerobically or combined with a photocatalysis pre-treatment. Borja et al. (1994a) showed a COD reduction of nearly 90% after aerobic biodegradation while Chatzisymeon et al. (2008) reached only a 60–65% when aerobic biodegradation was done after a photocatalysis pre-treatment, though the time needed for the photocatalysis treatment was at least twice the order of magnitude faster than the time needed for biological degradation.

#### Electro-Coagulation

Electro-coagulation is an electrochemical method with a sacrificial anode which is dissolved into the wastewaters in order to generate active coagulant precursors. The main precursors used are aluminum or iron cations which react with negatively charged particles present in the wastewaters (García-García et al., 2011).

The application of electro-coagulation technology as a pre-treatment for wastewaters from green table olive processing was evaluated by García-García et al. (2011). Under the best conditions (20–25°C; 50 min; 25 mA/cm^2^), 40% COD removal was obtained as well as the elimination of most of the phenol contents (78–87%) and color. pH was also neutralized (6.5–7.0). Across the several electrode combinations evaluated it was concluded that using Al in the anode and Fe in the cathode the COD, phenol and color concentrations were reduced faster than without them.

García-García et al. (2011) also studied the chemical reactions occurring on the electrode surfaces and in the bulk solution. They concluded that the pH growth was linked to the hydroxyl radicals liberated by the action of organic acids present in the wastewaters with the hydroxyl compounds of aluminum and iron after oxidation in the electrodes. Furthermore, the total consumption electricity spent was estimated at 0.76 kW h/m^3^_wastewater_, and the aluminum loss in the anode was 2.15 g/m^3^_wastewater_.

This technology has been successfully proven for color and the removal of colloidal particles, although a relatively low COD removal of 40% was reported (García-García et al., 2011).

#### Wet Air Oxidation

Wet air oxidation is a thermochemical AOP. The use of high temperature (200 – 320°C) and pressure (2 – 20 MPa) allow the water molecule to form hydroxyl radicals and other active oxygen species which react with organic matter producing highly oxidized compounds and eventually carbon dioxide and water (Levec and Pintar, 2007; Katsoni et al., 2008).

Rivas et al. (2001) treated diluted wastewaters from the washing and de-bittering processes of green table olives with ultrapure water (1:2). Wet air oxidation was carried out in batch mode with an initial air pressure of 1 MPa for 6–8 h. In addition, copper (II) sulfate was added as catalyst. The results showed that the higher the amount of copper added (50.8–419.4 mg/L), the higher the COD reduction. The influence of O_2_ partial pressure was also analyzed, and the results showed that the higher the pressure applied (3.0–7.0 MPa) the higher the COD removal, especially the phenol reduction, which reached 95%. A first-order kinetics related to COD removal was applied and the kinetic constant under the best conditions (7.0 MPa) was 3.97 ± 0.47⋅10^-5^ s^-1^. Temperature (170–210°C) was also modified in order to deduce its influence on COD removal, although no representative differences were detected. The influence of hydrogen peroxide (340–3400 mg/L) was also taken into account. Finally, it was concluded that the highest COD reduction (59.8%) was obtained at 180°C, with an oxygen partial pressure of 5 MPa and with 419.4 mg/L of Copper (II), and with these conditions phenol conversion was 76.8%. The best conditions for phenol conversion (94.9%) were at 180°C with an oxygen partial pressure of 5 MPa and 50.8 mg/L of Cu^+2^, while COD removal was 28.5%.

Furthermore, Rivas et al. (2001) evaluated the effect of a wet air oxidation treatment prior to aerobic degradation. Due to the negative effect of Cu^+2^ on biodegradability, most of the experiments carried out used wastewater with hydrogen peroxide which was further diluted with synthetic urban waters. Thus, the use of wet air oxidizes wastewater after 10 h and at 20°C, achieving a COD reduction of between 23.4 and 77.1%.

Wastewaters from black table olive fermentation processes were used by Katsoni et al. (2008) with the aim of evaluating the influence of initial substrate concentration (1240–5150 mg COD/L), operation time (30–120 min), temperature (140–180°C), initial pH (3–7) and H_2_O_2_ (500 mg/L) as an additional oxidant during wet air oxidation. Wet air oxidation was performed in an autoclave and pure O_2_ was fed continuously, the O_2_ partial pressure was maintained at 2.5 MPa. It was observed that across the different parameters evaluated, the operation time, the temperature and the initial pH presented a higher effect on COD removal, while initial COD, reaction time and temperature showed an important influence on phenol removal. Under the best conditions (initial COD 1240 mg O_2_/L; temperature of 180°C; pH 7; time 120 min and H_2_O_2_ 0 mg/L) phenol removal was complete, de-colorization reached 90% and COD reduction was 70%.

Therefore, the phenol and COD removals in these processes were clearly influenced by oxygen partial pressure, temperature and reaction time.

### Biological Treatments

Biological treatment processes utilize microorganisms to remove the organic matter contained in wastewaters. They can be classified into aerobic and anaerobic processes according to the type of microorganisms used and the operational conditions, i.e., presence or absence of oxygen. These processes have been widely and successfully applied for the treatment and purification of many high- and medium-organic content wastewaters. However, there are not many scientific works described in the literature related to the application of biological processes for treating table olive wastewater. This is because of its elevated content in recalcitrant and phenolic compounds, which are characterized by high toxicity and antimicrobial effect (Ayed et al., 2017). Moreover, the severe pH values, high salinity and unbalanced composition of TOPW may inhibit microbial growth and metabolism when biological treatment technologies are applied (Papadaki and Mantzouridou, 2016). **Tables 3–5** summarize the operating conditions, process efficiencies and benefits derived from the use of anaerobic, aerobic and combined anaerobic-aerobic processes, respectively, for the purification of these wastewaters.

**Table 3:** Summary of the operating conditions, process efficiencies and benefits derived from the use of anaerobic processes for TOPW treatment.

Wastewater type	Treatment	Operating conditions	Process efficiency	Benefits	Reference
Black olive wastewater	Anaerobic	CSTR reactor at mesophilic temperature (35°C). HRTs = 2.5–10 d	COD removal: 93%.	0.035 g VSS/g COD (low biomass yield coefficient) and 0.078 g COD/(g VSS⋅d) (specific rate of substrate removal for cell maintenance).	Borja et al., 1993
Black olive wastewater	Anaerobic	CSTR reactor at mesophilic temperature (35°C).	COD removal: 94.5% -92.6%	A decrease was observed in the biomass yield coefficient by 6 times and an increase in the specific rate of substrate uptake by 5 times.	Borja et al., 1994b
Black olive wastewater	Anaerobic	Batch reactors (with different microorganism immobilization supports) at mesophilic temperature (35°C).	Average COD removal: 95%.	Influence of the bacterial immobilization support on the methane yield, with values of 333 and 316 mL CH_4_/g COD for the reactors with sepiolite and bentonite, respectively.	Borja et al., 1992
Green table olive processing wastewater	Anaerobic	Batch mode at mesophilic temperature (35°C).	COD removal: 81–94%.	Mean methane yield coefficient: 270 mL CH_4_/g COD	Beltrán et al., 2008
Green olive de-bittering wastewater	Anaerobic	Reactors fed in a fill and draw mode at mesophilic temperature (35°). OLRs from 0.33 to 0.94 g COD/(L⋅d).	COD removal: 49%.	The process was severely inhibited.	Aggelis et al., 2001
De-bittering and washing effluent (DWE) with cattle manure (CM) and pig manure (PM)	Anaerobic co-digestion	Batch mode at mesophilic (35°C) and thermophilic temperatures (55°C).	VS removals: 65–73% (35°C) and 70–77% (55°C)	Co-digestion of TOPW with other substrates with different characteristics improve synergic effects between the microorganisms. Methane yields of between 250–300 mL CH_4_/g VS_added_ at 35°C and between 270 and 350 mL CH_4_/g VS_added_ at 55°C.	Zarkadas and Pilidis, 2011


**Table 4:** Summary of the operating conditions, process efficiencies and benefits derived from the use of aerobic processes for TOPW treatment.

Wastewater type	Treatment	Operating conditions	Process efficiency	Benefits	Reference
Black olive wastewater	Aerobic (activated-sludge system)	HRT: 10 h; SRT: 4–15 d.	COD and BOD removals: 92%.	The effluent COD concentration, specific maximum growth rate, and half-saturation constant were all dependent on the feed substrate concentration. No sludge-settling problems were detected.	Borja et al., 1994a
Green table olive wastewater	Aerobic (activated-sludge system)	HRT: 0.51–0.37 d, Dissolved oxygen: 2–3 mg/L.	COD removal: 75–85%.	Increasing the HRT from 0.37 to 0.51 days and the temperature from 10 to 32°C increased the efficiency of process. NaCl concentrations of up to 3% did not influence the COD removal efficiency of the process.	Brenes et al., 2000
Green olive de-bittering wastewater	Aerobic	Draw-and-fill mode reactor, temperature: 25°C, HRT: 10 d.	COD removal: 71.6–75.9%.	A COD/N/P ratio of approximately 100/5/1 is adequate to maintain satisfactory microbial activity in the culture.	Aggelis et al., 2001
Mixture of washing waters and de-bittering wastewaters (at a ratio 3:1 by volume)	Aerobic	Well-mixed batch reactor. Temperature: 28°C, air flow-rate: 50 L/h.	COD removal: 86%.		Benítez et al., 2002b
Green table olive wastewater	Aerobic	Batch reactor, temperature: 28°C.	COD removal: 49–67%.	Total phenolic compound removal varied between 92% and 100%.	Beltrán et al., 2008
Black olive wastewater	Aerobic	Shake-flask reactors, operating at 150 rpm and 20°C.	COD removal: 65%.	The biodegradation rate of the original effluent was three times higher than the oxidized one using TiO_2_ and hydrogen peroxide.	Chatzisymeon et al., 2008
Table olive processing wastewater	Aerobic	Suspended and attached growth reactors (trickling filters) operating with influent COD of between 5500 and 15000 mg/L	COD removals of 71.7 and 82.7% were achieved after 6 and 8 days of treatment respectively.	For a feed COD concentration of 5500 mg/L, the total phenolic compound removal was 67%.	Tatoulis et al., 2016


*SRT: solids retention time.*

**Table 5:** Summary of the operating conditions, process efficiencies and benefits derived from the use of anaerobic-aerobic treatment combinations for TOPW treatment.

Wastewater type	Treatment	Operating conditions	Process efficiency	Benefits	Reference
Green olive de-bittering wastewater	Anaerobic-aerobic	HRT: 50 d (anaerobic) and 5 d (aerobic).	COD removal: 83.8%	The successive anaerobic-aerobic treatment resulted in a lower amount of aerobic sludge and does not need a pH correction of the anaerobic or the aerobic influent.	Aggelis et al., 2001
Fermentation brines from table olive packaging industries	Anaerobic - aerobic	Two SBR working in parallel (SBR1 and SBR2). In SBR-1, the sludge was preliminarily acclimated to a high concentration of salt, while in SBR-2, the acclimatization of the sludge was made directly with TOPW.	COD removals: 88% (SBR1) and 73% (SBR2).	All phenols were completely removed from SBR-1 and SBR-2.	Ferrer-Polonio et al., 2015
Fermentation brines from table olive packaging industries	Anaerobic - aerobic	Different anaerobic-aerobic ratios were tested.	COD removal: 82.3%	The best anaerobic/aerobic ratio was 0/22. For a ratio of 8/14 the reactor consumed much less energy.	Ferrer-Polonio et al., 2016a
Fermentation brines from table olive packaging industries	Anaerobic - aerobic	SBRs with an optimal COD/N/P ratio of 250/5/1.	COD removal: 80%.		Ferrer-Polonio et al., 2016b
Fermentation brines from table olive packaging industries	Anaerobic - aerobic	SBRs.	COD removal: 80%.	It was observed that the increase in hydraulic retention had an effect on the decrease in organic matter.	Ferrer-Polonio et al., 2017a
Fermentation brines from table olive packaging industries	Anaerobic - aerobic	SBRs with ultrafiltration and nanofiltration.	The total integrated process gave effluents with COD < 125 mg/L.	The turbidity and the characteristic color of this type of wastewater were completely removed.	Ferrer-Polonio et al., 2017b
Table olive processing wastewater	Anaerobic - aerobic	SBRs.	COD removal: 80%.	TOPW salinity increased the reactor’s conductivity over time.	Soler-Cabezas et al., 2017


#### Anaerobic Treatment

Anaerobic digestion is a complex biological process in which organic raw substances are transformed to biogas, a mixture of methane (50–75%) and carbon dioxide (30–40%), and traces of other minor components(H_2_, H_2_S, etc.) by different groups of microorganisms which are sensitive to or completely inhibited by oxygen (Borja and Rincón, 2017). Using anaerobic digestion, it is possible to transform wastewaters from many industries into profitable by-products, mainly biogas, a useful fuel that may be used to provide heat, electrical power or combustible for transport. The transformation of organic matter to biogas occurs through four steps: hydrolysis, acidogenesis, acetogenesis and methanogenesis. The hydrolysis stage degrades both insoluble organic substances and high molecular weight compounds such as lipids, polysaccharides and proteins into soluble organic compounds. In a second stage, volatile fatty acids are generated by acidogenic or fermentative bacteria, as well as NH_3_, CO_2_, H_2_S, and other intermediate compounds. The third stage is acetogenesis. In this step the higher organic acids and other compounds produced by acidogenesis are further digested by acetogens to generate acetic acid, CO_2_ and H_2_. Finally, the fourth stage, or methanogenesis, produces methane. Two groups of methanogenic microorganisms produce methane: the first group converts acetate into methane and carbon dioxide (aceticlastic methanogens) and the second group uses hydrogen as electron donors and CO_2_ as acceptor to produce methane (hydrogenotrophic methanogens) (Borja and Rincón, 2017). The main advantages of the anaerobic digestion process over other forms of waste treatment are: quite a high degree of purification with high-organic-load feeds can be achieved; up to 90% reduction in space requirements; a combustible biogas is generated (around 31 m^3^ of methane per 100 kg of COD, with a maximum energetic value of 108 kWh in electric energy or 308 kWh in heat); the generation of biogas enables the process to produce energy; no use of fossil fuels for treatment (saving about 0.5–1 kWh per kg of organic matter); lower biomass sludge is produced in comparison to aerobic treatment processes, specifically a decrease in excess sludge production by 90% is detected; the sludge generated (digestate) is very stable and is an enhanced fertilizer in terms of both its availability to plants and its rheology; fewer nutrient requirements are necessary with optimum C:N:P of 100:0.5:0.1, which is 10% of the nutrient demand for the adequate development of the aerobic process.

An early study demonstrated the suitability of the anaerobic digestion process to treat the wastewater generated in the manufacturing of black olives (pH: 9.1; COD: 2.5 g/L; TS: 2.3 g/L) (Borja et al., 1993). This process was performed in a 1-l, continuous flow, completely mixed reactor operating at 35°C. The reactor performed satisfactorily at hydraulic retention times in the range of 2.5–10 days removing more than 93% of the initial COD in all cases. The macroenergetic parameters of this system were determined using Guiot’s kinetic model and were found to be 0.035 g VSS/g COD (biomass yield coefficient) and 0.078 g COD/(g VSS⋅d) (specific rate of substrate removal for cell maintenance). This study also showed that the rate of substrate removal was related with the concentration of biodegradable substrate through an equation of the Michaelis-Menten type (Borja et al., 1993). An additional research of the anaerobic digestion of black olive wastewater at the above-mentioned operational conditions using increasing influent substrate concentrations in the range of 1.4–4.4 g O_2_/L showed a decrease in the biomass yield coefficient by 6 times and an increase in the specific rate of substrate uptake by 5 times. This fact may be attributed to the higher mineral (sodium and potassium ions) solids and phenolic compound concentrations present in the most concentrated influents (Borja et al., 1994b). Anaerobic digestion experiments on black olive wastewater in batch mode at mesophilic temperature (35°C) revealed the influence of the bacterial immobilization support on the methane yield, with values of 333 and 316 mL CH_4_/g COD for the reactors with Sepiolite and Bentonite, respectively, as microorganism support. Average COD removal efficiencies of 95% were achieved, although a gradual decrease in the specific rate constant was observed for substrate concentrations higher than 1 g COD/L, showing the occurrence of an inhibition process (Borja et al., 1992).

The anaerobic digestion process of green table olive processing wastewater was also studied at mesophilic temperature (35°C) in batch mode (Beltrán et al., 2008). COD removal efficiencies between 81 and 94% were obtained for influent substrate concentrations in the range of 0.6–3.0 g COD/L, with a mean methane yield coefficient of 270 mL CH_4_/g COD. However, the global kinetic constants, obtained with the modified Monod model, diminished from 0.067 to 0.014 h^-1^ when the influent concentration increased to between the above-mentioned values, indicating that some inhibition effects took place by the phenolic substances contained in the wastewater (Beltrán et al., 2008).

Aggelis et al. (2001) also evaluated the mesophilic anaerobic digestion process of green olive de-bittering wastewater in reactors fed in a fill and draw mode and in continuously-stirred tank reactors (CSTR), achieving a 49% maximum efficiency of organic matter reduction with a polyphenol removal of about 12%, when the reactor operated at hydraulic retention times (HRTs) in the range of 50–25 days and organic loading rates from 0.33 to 0.94 g COD/(L⋅d). This process was severely inhibited as suggested by the low and restricted COD removal efficiency, volatile fatty acid accumulation and low methane production.

High COD removals (80–95%) and methane yields were attained in anaerobic digestion processes of these wastewaters. However, these processes are sometimes inhibited by the high content of phenolic compounds and high pH and salinity present in some of these wastewaters.

#### Anaerobic Co-digestion of TOPW With Other Wastes

An alternative considered by some researchers to minimize the difficulties and overcome the inhibition found in the anaerobic digestion of TOPW is its co-digestion with other wastes. Co-digestion with other substrates with different characteristics would allow to compensate toxicity and nutrient imbalance and to improve synergetic effects between the microorganisms (Zarkadas and Pilidis, 2011). For instance, batch experiments carried out with different mixtures of table olive de-bittering and washing effluents (DWE) with cattle manure (CM) and pig manure (PM) resulted in ultimate methane yields of between 250 and 300 mL CH_4_/g Volatile Solids_added_ at a mesophilic temperature of 35°C and between 270 and 350 mL CH_4_/g Volatile Solids_added_ at a thermophilic temperature 55°C. The highest methane production for the thermophilic temperature was achieved for a combination of wastewater containing 35% CM, 35% PM and 30% DWE (C/N ratio of 17.6), while for mesophilic digestion the highest methane yield was found for a mixture of 50% CM, 25% PM and 25% DWE (C/N ratio of 19.3). In these cases, no inhibition was observed since there was a small lag-adaptation of 3 days at the beginning of the process. In addition, no volatile fatty acid accumulation was observed, showing that the reactors were not operating under stress-overloading conditions (Zarkadas and Pilidis, 2011).

No inhibition and better stability were observed in the co-digestion processes of DWE with other substrates with different characteristics compared with the single anaerobic digestion process of the DWE.

#### Aerobic Treatments

The effectiveness of the aerobic treatments for removing the polluting load of the TOPW varies considerably depending on the class of microorganisms, organic substances to be removed and the environmental factors that influence process performance. In aerobic treatments the microorganisms oxidize the dissolved and particulate carbonaceous organics into simpler compounds and new sludge (Ayed et al., 2017).

A first study revealed that both natural and diluted black olive wastewaters (700–2200 mg COD/L) were easily purified by a completely mixed activated-sludge treatment system (Borja et al., 1994a). At least 92% of the COD and BOD were reduced in this system at an HRT of 10 h and solid retention time of 4–15 days. It was observed that the effluent COD concentration, specific maximum growth rate, and half-saturation constant were all dependent on the influent substrate concentration. The multiple-substrate model of Adams et al. (1975) allowed to predict adequately the effluent COD under variable influent COD concentrations. Finally, no sludge-settling problems were detected in this aerobic treatment (Borja et al., 1994a).

Another study on the purification of green table olive wastewaters by an activated-sludge system showed COD removal efficiencies in the range of 75–85%, when the reactor operated with influent COD concentrations in the range of 2500–3250 mg/L, HRTs of between 0.51 and 0.37 days (constant cellular retention time: 3.32 days) and with dissolved oxygen varying between 2 and 3 mg/L (Brenes et al., 2000). COD removal was mainly due to the reduction in organic acids and the ethanol present in the wastewater. On the contrary, only a low portion of polyphenols was removed. These polyphenols, especially those in a polymerized state, were not removed and were responsible for the color of the solutions and the residual measured COD. The substrate removal model proposed by Grau was applied to consider the effect of influent-substrate concentration on the effluent COD concentration, with the kinetic constant obtained at 9.8 days^-1^. Increasing the HRT from 0.37 to 0.51 days and the temperature in the range of 10–32°C augmented the efficiency of the sludge activated process, obtaining effluent COD values of 200–300 mg/L in all cases studied. Concentrations of NaCl up to 3% did not affect the COD removal efficiency of the process, although the sludge volume index was higher than 200 cm^3^/g (Brenes et al., 2000).

An aerobic treatment of green olive de-bittering wastewater was also evaluated with an influent COD of 16500 mg/L and polyphenol concentration of 1350 mg/L (Aggelis et al., 2001). In this study, a laboratory 1-L (useful) volume stirred draw-and-fill aerobic reactor was used. The reactor operated at a temperature of 25°C, using an HRT of 10 days and organic loading rates in the range of 1.6–2.3 g COD/(L⋅d) with pH adjustment of the wastewater to a value below 8.5. Na_2_HPO_4_ and urea were added as nutrients in order to maintain a COD/N/P ratio of approximately 100/5/1, which is appropriate to maintain a high microbial activity in the culture (Aggelis et al., 2001). This aerobic treatment was more effective than its anaerobic digestion, resulting in a degradation efficiency of 71.6–75.9%. However, it hardly affected the polyphenolic compound content, with the additional disadvantages of the requirement for pH correction of the influent and the high generation of biomass due to the aerobic metabolism (Aggelis et al., 2001).

The aerobic treatment of a mixture of washing waters and de-bittering wastewaters (at a ratio 3:1 by volume) was assessed by Benítez et al. (2002b). This research was performed in a 1-l well-mixed batch reactor at a constant temperature of 28°C with an air flow-rate of 50 L/h, with an influent COD concentration of 3.85 g/L. COD and BOD diminished continuously with reaction time. The overall COD reduction was 86% at the end of the experiment (7 days). The biomass variation agreed well with the typical growth-cycle phases for batch cultures: acclimation stage (lag phase), increase in the biomass concentration (exponential growth phase), maximum size of population (stationary stage) and decline in cell numbers (death phase) (Benítez et al., 2002b).

More recently, the aerobic biodegradation of green table olive wastewater was also investigated by Beltrán et al. (2008). A batch reactor was used in this research, which operated at 28°C, with influent substrate concentrations and initial biomass concentrations in the range of 9.5–41.6 g COD/L and 0.2–2.2 g VSS/L, respectively. The total polyphenolic concentration present in the wastewater was 3.1 g caffeic acid/L. Total COD removal efficiencies ranged between 49 and 67%, while total phenolic compound removal varied between 92 and 100%. A kinetic study allowed for determining the cellular yield coefficient (*Y_x/s_* = 0.057 g VSS/g COD) and the kinetic constant of cellular death phase (*k_d_* = 0.16 d^-1^) (Beltrán et al., 2008).

An aerobic treatment of black olive wastewater (COD: 40 g/L; total phenols: 3.6 g/L) performed in shake-flask reactors, operating with non-acclimated activated sludge at 150 rpm and 20°C revealed that this wastewater was partially biodegradable aerobically (Chatzisymeon et al., 2008). After 16 days of treatment in batch mode, COD removal was 65%. Moreover, it was found that biosorption was always less than 20%. Therefore, the observed COD removal after the mentioned time of incubation was attributed mainly to the biodegradation of the organic content of the effluent by the non-acclimated sludge. In addition, the biodegradation rate of the original effluent was three times greater than the oxidized one using TiO_2_ and hydrogen peroxide (Chatzisymeon et al., 2008).

In a recent study, table olive processing wastewaters were cleansed in aerobic biological reactors using native microorganisms originating from these wastewaters (Tatoulis et al., 2016). The aerobic biological processes were performed in suspended and attached growth reactors (trickling filters) using different feed substrate concentrations of 5500, 7500, and 15000 mg COD/L. Two different operating modes were studied to determine the optimum performance of the filter, i.e., batch and SBR with recirculation. In the batch suspended-growth flask reactors, COD removals of 71.7 and 82.7% were reached after 6 and 8 days of treatment at influent concentrations of 5500 and 7500 mg COD/L, respectively. In addition, for an initial COD concentration of 5500 mg/L, the total phenolic compound removal was 67%, while for the higher influent concentrations of 7500 and 15000 mg COD/L, phenolic compounds decreased to 63 and 57%, respectively (Tatoulis et al., 2016).

Except for olive de-bittering wastewaters, which are characterized by their high pH, anaerobic treatments showed higher COD and phenolic compound removals compared with those obtained in the aerobic processes.

#### Anaerobic and Aerobic Treatment Combinations

The aerobic treatment of the anaerobic digestion effluent of green olive de-bittering wastewater resulted in COD and polyphenolic compound removals of 74 and 19.6%, achieving an overall depletion of 83.8 and 28%, respectively, operating at HRTs of 50 days (anaerobic stage) and 5 days (aerobic stage) (Aggelis et al., 2001). Most likely, the anaerobic pre-treatment of the original green olive wastewater hydrolyzes polyphenolic compounds, giving a more readily biodegradable compound under aerobic conditions. In addition, the successive anaerobic-aerobic treatment resulted in a lower amount of aerobic sludge and does not need a pH correction of the anaerobic or the aerobic influent (Aggelis et al., 2001).

Another option is the use of SBRs. SBRs can be defined as a system of activated sludge, the functioning of which is based on the sequence of aerobic and anaerobic treatment phases. With the combination of these two treatments, organic matter, nitrogen and phosphorus can be removed simultaneously (Pavšelj et al., 2001).

With the introduction of the anaerobic phase the release of phosphorus occurs with part of the microorganisms; in the aerobic phase the nitrification, consumption of oxygen and phosphorus take place; while the denitrification occurs in the next anoxic phase (Cárdenas et al., 2006).

Ferrer-Polonio et al. (2015) studied the effect of various start-ups of SBRs for treating TOPW. In SBR-1, the sludge was preliminarily acclimated to a high concentration of salt, but not to a high concentration of phenols. While in SBR-2, the acclimatization of the sludge was made directly with TOPW. They reported that salinity promoted the population of 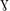-proteobacteria at the expense of other microorganisms. It was also observed that the SBR-1 had more operational problems consisting of a higher de-flocculation than SBR-2 which led to high turbidity values in the effluent, and finally the organic matter removal in this reactor was lower than the organic matter reduction achieved in SBR-2. All phenols were completely eliminated from SBR-1 and SBR-2, concluding that the concentration of phenols contained in TOPW was not an inhibitory concentration for the bacteria present in these bioreactors.

An SBR treating TOPW was used by Ferrer-Polonio et al. (2016a). They tested different anaerobic/aerobic ratios and found that the ratio that best adapted to this type of water was 0/22, where they achieved a COD reduction of 82.3 and 77.9% for total phenols. However, the ratio of 8/14 was determined as the optimal ratio, since the reduction in nutrients was very similar and working in this way the reactor consumed much less energy.

Ferrer-Polonio et al. (2016b) also treated TOPW with SBR with the aim of reducing hydraulic retention times by adding extra nutrients to TOPW and discovered that a COD/N/P ratio of 250/5/1 was optimal for the biological process to work efficiently. During this experiment COD was reduced by up to 80%. They also studied the population of bacteria in their reactors, observing that the main bacteria were 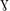-proteobacteria.

In another study applying the SBR technology on TOPW, the effect of alternating aerobic/anaerobic treatment on the protist population was assessed (Ferrer-Polonio et al., 2017a). During this study the authors observed that the increase in hydraulic retention not only had an effect on the decrease in organic matter, but also favored the population of ciliates against flagellates.

In another recent study by Ferrer-Polonio et al. (2017b) related to the treatment of TOPW, a mixed SBR technology with ultrafiltration and nanofiltration was evaluated. It was reported that only with the SBR 80% of the organic matter and 71% the total phenol concentration were removed, but with the addition of ultrafiltration and nanofiltration, the COD was finally less than 125 mg/L, with a final COD removal of 45.9 ± 1.9% (the high salinity could be responsible for this lower COD reduction). In addition, the turbidity and the characteristic color of this type of wastewater were completely removed.

Soler-Cabezas et al. (2017) also applied the SBR technology to purify TOPW, finding that this system was able to reduce 80% COD and 76% total phenol concentration. The main problem was that TOPW salinity increased the reactor’s conductivity over time.

The combined anaerobic-aerobic processes allow to achieve higher COD removals compared with single anaerobic or aerobic processes, which result relevant and very efficient for wastewaters with inhibitory compounds (i.e., olive de-bittering wastewaters).

### Bioremediation Technologies

#### Use of Microalgae

Microalgae and cyanobacteria are photosynthetic microorganisms able to produce oxygen which can be used for the oxidation of organic matter and NH_4_^+^, saving in aeration costs; while the autotrophic and heterotrophic growth of algal and bacterial biomass lead to higher nutrient recoveries. The recent worldwide interest in the cultivation of microalgae for energy purposes, together with the need for environmentally more sustainable wastewater treatment technologies, have made microalgae wastewater treatment processes a promising alternative from economic and environmental points of view (Chinnasamy et al., 2014). In addition, the algal biomass produced can be a valuable raw material for the generation of bioenergy, biofertilizers and other valuable products.

Although the use of microalgae to treat wastewaters is widespread, it has hardly been used to treat TOPW. However, good results have been found by Serrano et al. (2017), who found removals of 69.1, 50.9, 54.3 and 71.85% for TOC, TSN (total soluble nitrogen), phosphate and total phenols respectively, when growing *Nannochloropsis gaditana* in TOPW diluted at 80%.

#### Use of Fungi

The treatment of wastewaters using fungi is widespread, especially nowadays when there are several studies about the ability of fungi to produce pharmaceutical products from water (Lucas et al., 2018).

Kyriacou et al. (2005) combined a biological process using *Aspergillus* strain with BDD treatment in the presence of hydrogen peroxide as a treatment for de-bittering and washing wastewaters. They reported 86 and 65% COD and total phenol removals, respectively, after the biological treatment. An improvement in the reduction of organic matter and total phenols was observed after the electrochemical treatment, achieving a total reduction of 98% for both organic matter and phenols.

Similar results have been reported by Lasaridi et al. (2010) using *Aspergillus niger* on fresh de-bittering wastewater and washing water. They used the main table olive de-bittering wastewater with NaOH and an alternative with KOH using dilutions of 100, 85, 70, 55, and 40%. All these wastewaters were inoculated with *Aspergillus niger*. These studies showed COD removal efficiencies in the range of 60–87% for NaOH and 50–87% for the KOH treatment (Lasaridi et al., 2010).

Different fungi strains were isolated from brine wastewaters by Crognale et al. (2012) with the aim of producing extracellular phenoloxidases. A total of 20 strains were isolated, although only two of them were significant effective in producing laccases and Mn-peroxidase, when grown under saline conditions (0–10% NaCl). *Citeromyces matritensis* (syn. *Candida globose*) and *Aspergillus fumigatus* 6C2 decreased the phenolic compounds of TOPW by up to 82.3%.

The *Geotrichum candidum* strain was used by Ayed et al. (2016) to study its effect on color reduction in de-bittering and washing wastewaters. A significant improvement in color reduction was observed when the growth of the fungus remained constant. The extracellular peroxidases of *G. Candidum* have been able to effectively reduce phenol content in TOPW, which is responsible for its coloration. The main phenols found, i.e., coumaric acid, oleuropein, tyrosol and vanillic acid have been reduced by more than 55% in all cases. During this treatment COD, color and total phenols decreased by 71, 63, and 60%, respectively.

The use of fungi for the treatment of TOPW is mainly focused on reducing the organic matter in general and particularly phenol contents with promising results. Moreover, these microorganisms allow obtaining valuable products (i.e., enzymes) although they need a pH regulation in the wastewater.

**Table 6** summarizes the operating conditions, process efficiencies and benefits derived from the use of microalgae and fungi for treatment and/or re-use of these wastewaters.

**Table 6:** Summary of the operating conditions, process efficiencies and benefits derived from the use of bioremediation processes (microalgae and fungi) for TOPW treatment.

Wastewater type	Treatment	Operating conditions	Process efficiency	Benefits	Reference
Table olive processing wastewater	Microalgae growth	*Nannochloropsis gaditana* TOPW diluted at 80%.	TOC removal: 69.1%. TSN removal: 50.9%. phosphate removal: 54.3%.	Nutrient recovery and algal biomass production that can be used for bioenergy production	Serrano et al., 2017
De-bittering and washing wastewater	Biological and electrochemical treatment	*Aspergillus* strain	Biological treatment: COD removal: 86%.	High organic matter reduction	Kyriacou et al., 2005
De-bittering and washing wastewater	Biological treatment	*Aspergillus niger.* NaOH and KOH treatment in water diluted 100, 85 70, 55, and 40%.	NaOH treatment; COD removal: 60–87%. KOH treatment; COD removal: 50–87%	High organic matter reduction	Lasaridi et al., 2010
Brine wastewater	Biological treatment	*Citeromyces matritensis* and *Aspergillus fumigates* 6C2.	Total phenol reduction: 82.3%	Extracellular phenoloxidases production and high organic matter reduction	Crognale et al., 2012
De-bittering and washing wastewater	Biological treatment	*Geotrichum candidum*	COD reduction: 71%.	High organic matter reduction	Ayed et al., 2016


*TSN: total soluble nitrogen.*

### Combination of Different Treatments

Membrane bioreactors (MBRs) are reactors which contain an active sludge and different types of membranes. These reactors have received great attention in the last decade due to significant cost reductions, which leads to an increase in the use of MBR systems for treating wastewater. The main advantages of new MBR technologies are: low space requirement, flexible configurations, stability, and elimination of the problems associated with the sedimentation of the sludge. However, there are few studies using MBR technology for the treatment of TOPW, despite the above-mentioned advantages.

Patsios et al. (2016) used activated sludge from a municipal wastewater treatment plant to activate the membrane. Then, the membrane was gradually acclimated to a high salinity substrate and, finally, TOPW was used directly. A TOC removal efficiency of 91.5% and an efficiency of total phenol removal of 82.8% were reported. Despite this high elimination of phenols, the resulting waters continued to have the problem of being colored.

Another recent study using MBRs for the treatment of TOPW was carried out by Soler-Cabezas et al. (2017). The main drawback reported in this study was the high salinity of the TOPW, which was finally eliminated, causing an increase in salinity in the reactor. On the other hand, the authors stated that this type of treatment, as a low energy cost process, was capable of removing up to 80% of COD.

### Other Uses

#### Irrigation

Agriculture consumes up to 70% fresh water. Many Mediterranean countries are suffering great periods of drought, so the reuse of industrial waters for agriculture would solve a major problem of water shortage, in addition to directly providing nutrients to the soil (Libutti et al., 2018).

Murillo et al. (2000) investigated the application of TOPW for the irrigation of olive trees. However, they found that in just 15 days of using TOPW as irrigation water, a decrease in leaf water potential and a stomatal conductance to water and photosynthesis were observed.

In addition, a reduction in the nitrogen load of the leaf was also reported. The authors assumed that this type of wastewater is totally unsuitable for agricultural proposes due to its characteristics and above all, to its high salinity concentrations.

#### Extraction and Recovery of Added-Value Products

Phenols are strong antioxidants which are difficult to synthesize and can be easily extracted from the fruit of the olive. The effect of three commercial membranes to concentrate phenols from TOPW was examined by Kiai et al., (2014). The use of a direct contact membrane distillation process, regardless of the membrane type tested, showed separation coefficients which were greater than 99.5%. However, membrane TF450 at 70°C gave the highest concentration factors. In addition, the most resistant membrane to the fouling phenomenon was TF200, with the lowest pore size.

The washwaters from Spanish-style green olive (Hojiblanca type) processing have also been treated by combined fermentation and evaporation systems for the recovery of phenols (Brenes et al., 2004). The fermentation step was conducted on a pilot plant scale (500 L) and stored for 9 months. These results showed that there was no significant difference when wastewaters were acidified to pH 5 and inoculated with *Lactobacillus pentosus* or acidified to pH 3.4 and without inoculation. The fermentation process reduced the COD by up to 20%. After the evaporation step (rotary evaporator under vacuum at 65°C) a large amount of high-value compounds were found in the concentrate (lactic acid and hydroxytyrosol at concentrations up to 123.7 ± 1.6 g/dm^3^ and 36.4 ± 1.6 g/dm^3^, respectively). Furthermore, it was observed that the fermentation step without any pH adjustment was mainly conducted by putrefactive bacteria (*Enterobacteriaceae* family), which produce a large amount of gas and strong off-odors.

An investigation on wastewaters from table olive brine reported hydroxytyrosol and tyrosol concentrations of 690 and 98 mg/g dry weight extract, respectively (Bouaziz et al., 2008). These authors also tested the phenol antioxidant power against 2 human cancer cell lines with promising results.

In another study about Megaritike, a Greek style olive oil and table olive, the authors were able to concentrate and recover phenols with an adsorption resin. They found that the main phenols present in this kind of TOPW were hydroxytyrosol-4-*O*-glucoside, 11-methyl-oleoside, hydroxytyrosol and tyrosol (Mousouri et al., 2014).

The extraction and recovery of added-value products from TOPW would allow reducing the operating costs of other treatment processes, and at the same time, this procedure would result in an improvement in the efficiency of biological processes avoiding inhibitory processes.

## Conclusion and Future Trends

The legislation in different countries regarding environmental issues is becoming increasingly stricter. In order to achieve a decrease in environmental impact, the segregation of the effluents to be treated would be necessary as proposed previously by different researchers. Moreover, the decrease in the amount of materials used (NaOH, NaCl, H_2_O, etc.), the use of low concentration lyes, the reuse of fermentation brines would lead to a decrease in the polluting content and the high volumes of resulting wastewater. Different studies have been developed on the treatment and management of table olive wastewaters, but the reality is that these treatments are not applied to a great extent at the industrial level. The problem continues to be very serious and further research into new approaches to the problem is needed. Among these approaches, integrated purification processes combining a first step of chemical oxidation, with stronger advanced oxidation methods (i.e., Ozonation and Electrochemical treatments with BDD), with a second biological step are a promising alternative. Another challenge to be considered would be the management of the sludge that is produced during the biological treatment: transformation to compost and the use of the biomass generated as renewable energy sources. In addition, different combined fermentation and evaporation systems should be further studied in order to maximize phenol recovery, given the high value of these compounds as antioxidant agents. All these considerations will be focused on to achieve a circular economical model and to obtain the desired zero waste, which is of great interest for European table olive producing countries.

## Author Contributions

BR-L, DL-C, MF-R, and RB-P did the bibliographic search and wrote the full manuscript. BR-L planned the index and structure of the paper, coordinated the work and the final manuscript.

## Conflict of Interest Statement

The authors declare that the research was conducted in the absence of any commercial or financial relationships that could be construed as a potential conflict of interest.
